# (*E*)-1-{4-[Bis(4-bromo­phen­yl)meth­yl]piperazin-1-yl}-3-(4-methyl­phen­yl)prop-2-en-1-one

**DOI:** 10.1107/S1600536812003820

**Published:** 2012-02-04

**Authors:** Yan Zhong, XiaoPing Zhang, Bin Wu

**Affiliations:** a School of Chemistry and Chemical Engineering, Southeast University, Sipailou No. 2 Nanjing, Nanjing 210096, People’s Republic of China; b Centre of Laboratory Animal, Nanjing Medical University, Hanzhong Road No. 140 Nanjing, Nanjing 210029, People’s Republic of China; c School of Pharmacy, Nanjing Medical University, Hanzhong Road No. 140 Nanjing, Nanjing 210029, People’s Republic of China

## Abstract

In the title compound, C_27_H_26_Br_2_N_2_O, the piperazine ring adopts a chair conformation with the N—C bonds in equatorial orientations. The C=C double bond has an *E* configuration. The dihedral angle between the bromo­benzene rings is 83.0 (4)°. In the crystal, inversion dimers linked through pairs of C—H⋯O hydrogen bonds generate *R*_2_^2^(10) loops.

## Related literature

For a related structure and background to cinnamic acid derivatives, see: Teng *et al.* (2011 ▶); Zhong *et al.* (2012 ▶). For further synthetic details, see: Wu *et al.* (2008 ▶).
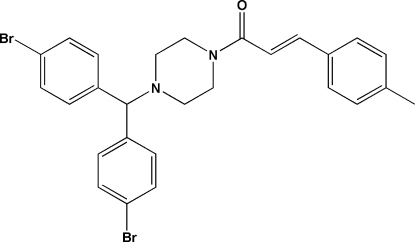


## Experimental

### 

#### Crystal data

C_27_H_26_Br_2_N_2_O
*M* *_r_* = 554.32
Monoclinic, 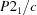

*a* = 10.050 (2) Å
*b* = 11.622 (2) Å
*c* = 21.259 (4) Å
β = 101.72 (3)°
*V* = 2431.3 (8) Å^3^
*Z* = 4
Mo *K*α radiation
μ = 3.36 mm^−1^
*T* = 293 K
0.20 × 0.10 × 0.10 mm

#### Data collection

Enraf–Nonius CAD-4 diffractometer
Absorption correction: ψ scan (North *et al.*, 1968 ▶) *T*_min_ = 0.553, *T*_max_ = 0.730
4720 measured reflections
4452 independent reflections
1998 reflections with *I* > 2σ(*I*)
*R*_int_ = 0.097
3 standard reflections every 200 reflections intensity decay: 1%

#### Refinement

*R*[*F* ^2^ > 2σ(*F* ^2^)] = 0.075
*wR*(*F* ^2^) = 0.155
*S* = 1.00
4452 reflections
289 parameters
H-atom parameters constrained
Δρ_max_ = 0.34 e Å^−3^
Δρ_min_ = −0.45 e Å^−3^

### 

Data collection: *CAD-4 EXPRESS* (Enraf–Nonius, 1989 ▶); cell refinement: *XCAD4* (Harms & Wocadlo, 1995 ▶); data reduction: *XCAD4*; program(s) used to solve structure: *SHELXS97* (Sheldrick, 2008 ▶); program(s) used to refine structure: *SHELXL97* (Sheldrick, 2008 ▶); molecular graphics: *SHELXTL* (Sheldrick, 2008 ▶); software used to prepare material for publication: *SHELXL97*.

## Supplementary Material

Crystal structure: contains datablock(s) I, global. DOI: 10.1107/S1600536812003820/hb6616sup1.cif

Structure factors: contains datablock(s) I. DOI: 10.1107/S1600536812003820/hb6616Isup2.hkl

Supplementary material file. DOI: 10.1107/S1600536812003820/hb6616Isup3.cml

Additional supplementary materials:  crystallographic information; 3D view; checkCIF report

## Figures and Tables

**Table 1:** Hydrogen-bond geometry (Å, °)

*D*—H⋯*A*	*D*—H	H⋯*A*	*D*⋯*A*	*D*—H⋯*A*
C20—H20*A*⋯O^i^	0.93	2.55	3.445 (10)	162

Symmetry code: (i) 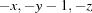.

**Fig. 1.**
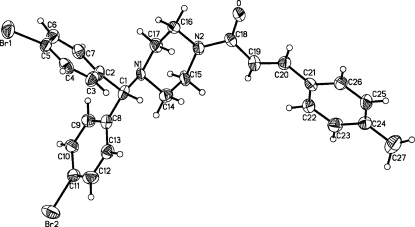
The molecular structure of (I) with displacement ellipsoids for non-H drawn at 70% probability level.

**Fig. 2.**
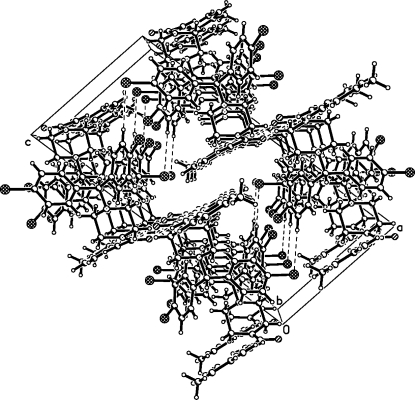
Packing diagram of the title compound.

## supplementary crystallographic information

### Comment

As a continuation of our study of cinnamic acid derivatives (Teng *et al.*, 2011; Zhong *et al.*, 2012), we present here the title compound (I). In (I) (Fig. 1), all bond lengths and angles are normal and correspond to those observed in related compounds (Teng *et al.*, 2011; Zhong *et al.*, 2012). The molecule of (I) exists an E configulation with respect to the C19=C20 ethene bond [1.317 (10)]. The piperazine ring adopts a chair conformation with puchering parameters Q = 0.574 (8), Theta = 5.0 (7), Phi = 2(10). In the crystal, molecules are linked by C—H···O hydrogen bonds to form inversion dimers.

### Experimental

The synthesis follows the method of Wu *et al.* (2008). The title compound was prepared by stirring a mixture of (*E*)-3-(4-methylphenyl) acrylic acid (0.649 g; 4 mmol), thionyl chloride (2 ml) and dichloromethane (30 ml) for 6 h at room temperature. The solvent was removed under reduced pressure. The residue was dissolved in acetone (15 ml) and reacted with 1-(bis(4-bromophenyl)methyl)iperazine (2.461 g; 6 mmol) in the presence of triethylamine (5 ml) for 12 h at room temperature. The resultant mixture was cooled. The solid, (*E*)-1-(4-(bis(4-bromophenyl)methyl)piperazin-1-yl) -3-(4-methylphenyl)prop-2-en-1-one obtained was filtered and was recrystallized from ethanol. Colourless blocks were grown from an ethanol:ethyl acetate (1:1) solution by slow evaporation at room temperature.

### Refinement

All non-hydrogen atoms were refined anisotropically. All hydrogen atoms were positioned geometrically with C—H distances ranging from 0.93 Å to 0.98 Å and refined as riding on their parent atoms with *U*ĩso~(H) = 1.2 or 1.5*U*~eq~ of the carrier atom.

### Crystal data

**Table:**

C_27_H_26_Br_2_N_2_O	*F*(000) = 1120
*M**_r_* = 554.32	*D*_x_ = 1.514 Mg m^−^^3^
Monoclinic, *P*2_1_/*c*	Mo *K*α radiation, λ = 0.71073 Å
*a* = 10.050 (2) Å	Cell parameters from 25 reflections
*b* = 11.622 (2) Å	θ = 9–13°
*c* = 21.259 (4) Å	µ = 3.36 mm^−^^1^
β = 101.72 (3)°	*T* = 293 K
*V* = 2431.3 (8) Å^3^	Block, colorless
*Z* = 4	0.20 × 0.10 × 0.10 mm

### Data collection

**Table:**

Enraf–Nonius CAD-4 diffractometer	1998 reflections with *I* > 2σ(*I*)
Radiation source: fine-focus sealed tube	*R*_int_ = 0.097
Graphite monochromator	θ_max_ = 25.4°, θ_min_ = 2.0°
ω/2θ scans	*h* = 0→12
Absorption correction: ψ scan (North *et al.*, 1968)	*k* = 0→14
*T*_min_ = 0.553, *T*_max_ = 0.730	*l* = −25→25
4720 measured reflections	3 standard reflections every 200 reflections
4452 independent reflections	intensity decay: 1%

### Refinement

**Table:**

Refinement on *F*^2^	Primary atom site location: structure-invariant direct methods
Least-squares matrix: full	Secondary atom site location: difference Fourier map
*R*[*F*^2^ > 2σ(*F*^2^)] = 0.075	Hydrogen site location: inferred from neighbouring sites
*wR*(*F*^2^) = 0.155	H-atom parameters constrained
*S* = 1.00	*w* = 1/[σ^2^(*F*_o_^2^) + (0.058*P*)^2^] where *P* = (*F*_o_^2^ + 2*F*_c_^2^)/3
4452 reflections	(Δ/σ)_max_ < 0.001
289 parameters	Δρ_max_ = 0.34 e Å^−^^3^
0 restraints	Δρ_min_ = −0.45 e Å^−^^3^

### Special details

**Table:**

Geometry. All e.s.d.'s (except the e.s.d. in the dihedral angle between two l.s. planes) are estimated using the full covariance matrix. The cell e.s.d.'s are taken into account individually in the estimation of e.s.d.'s in distances, angles and torsion angles; correlations between e.s.d.'s in cell parameters are only used when they are defined by crystal symmetry. An approximate (isotropic) treatment of cell e.s.d.'s is used for estimating e.s.d.'s involving l.s. planes.
Refinement. Refinement of *F*^2^ against ALL reflections. The weighted *R*-factor *wR* and goodness of fit *S* are based on *F*^2^, conventional *R*-factors *R* are based on *F*, with *F* set to zero for negative *F*^2^. The threshold expression of *F*^2^ > σ(*F*^2^) is used only for calculating *R*-factors(gt) *etc*. and is not relevant to the choice of reflections for refinement. *R*-factors based on *F*^2^ are statistically about twice as large as those based on *F*, and *R*- factors based on ALL data will be even larger.

### Fractional atomic coordinates and isotropic or equivalent isotropic displacement parameters (Å^2^)

**Table:**

	*x*	*y*	*z*	*U*_iso_*/*U*_eq_	
O	−0.0717 (5)	−0.3479 (4)	−0.0069 (3)	0.0630 (15)	
Br1	0.44042 (9)	0.37326 (8)	0.22953 (5)	0.0767 (4)	
N1	−0.0588 (5)	−0.0520 (5)	0.1495 (3)	0.0442 (15)	
C1	0.0218 (7)	−0.0187 (6)	0.2125 (4)	0.0487 (19)	
H1A	0.0653	−0.0883	0.2333	0.058*	
Br2	−0.32805 (9)	0.19580 (10)	0.38972 (4)	0.0806 (4)	
N2	−0.1598 (6)	−0.2188 (5)	0.0521 (3)	0.0545 (17)	
C2	0.1307 (7)	0.0670 (7)	0.2094 (4)	0.0470 (19)	
C3	0.2514 (8)	0.0676 (7)	0.2534 (4)	0.060 (2)	
H3A	0.2707	0.0050	0.2810	0.072*	
C4	0.3449 (8)	0.1542 (8)	0.2592 (4)	0.061 (2)	
H4A	0.4240	0.1510	0.2907	0.074*	
C5	0.3201 (8)	0.2465 (7)	0.2176 (4)	0.055 (2)	
C6	0.2044 (8)	0.2464 (7)	0.1691 (4)	0.054 (2)	
H6A	0.1901	0.3043	0.1382	0.065*	
C7	0.1108 (8)	0.1585 (7)	0.1676 (4)	0.057 (2)	
H7A	0.0306	0.1615	0.1368	0.068*	
C8	−0.0647 (7)	0.0317 (7)	0.2557 (4)	0.049 (2)	
C9	−0.1647 (8)	0.1108 (7)	0.2339 (4)	0.054 (2)	
H9A	−0.1798	0.1314	0.1908	0.065*	
C10	−0.2433 (8)	0.1612 (7)	0.2714 (4)	0.055 (2)	
H10A	−0.3092	0.2154	0.2548	0.067*	
C11	−0.2210 (8)	0.1285 (7)	0.3351 (4)	0.056 (2)	
C12	−0.1220 (8)	0.0515 (8)	0.3588 (4)	0.065 (2)	
H12A	−0.1051	0.0326	0.4022	0.079*	
C13	−0.0476 (8)	0.0019 (6)	0.3197 (4)	0.055 (2)	
H13A	0.0165	−0.0536	0.3364	0.066*	
C14	−0.1526 (7)	−0.1435 (7)	0.1605 (4)	0.057 (2)	
H14A	−0.2085	−0.1157	0.1896	0.069*	
H14B	−0.1009	−0.2089	0.1806	0.069*	
C15	−0.2449 (8)	−0.1819 (7)	0.0968 (4)	0.061 (2)	
H15A	−0.3026	−0.2449	0.1047	0.073*	
H15B	−0.3027	−0.1185	0.0783	0.073*	
C16	−0.0594 (8)	−0.1313 (7)	0.0440 (4)	0.064 (2)	
H16A	−0.1059	−0.0643	0.0229	0.077*	
H16B	−0.0008	−0.1616	0.0169	0.077*	
C17	0.0250 (8)	−0.0964 (7)	0.1075 (4)	0.058 (2)	
H17A	0.0757	−0.1624	0.1275	0.070*	
H17B	0.0897	−0.0380	0.1009	0.070*	
C18	−0.1507 (8)	−0.3269 (7)	0.0276 (4)	0.051 (2)	
C19	−0.2448 (8)	−0.4138 (7)	0.0431 (4)	0.055 (2)	
H19A	−0.3247	−0.3913	0.0552	0.066*	
C20	−0.2158 (8)	−0.5239 (7)	0.0396 (3)	0.052 (2)	
H20A	−0.1344	−0.5419	0.0276	0.063*	
C21	−0.2995 (7)	−0.6208 (7)	0.0530 (3)	0.0483 (19)	
C22	−0.4292 (8)	−0.6105 (7)	0.0646 (4)	0.060 (2)	
H22A	−0.4668	−0.5376	0.0657	0.072*	
C23	−0.5059 (9)	−0.7074 (8)	0.0748 (4)	0.064 (2)	
H23A	−0.5935	−0.6976	0.0819	0.076*	
C24	−0.4521 (9)	−0.8183 (8)	0.0743 (4)	0.059 (2)	
C25	−0.3214 (9)	−0.8263 (7)	0.0645 (4)	0.066 (2)	
H25A	−0.2823	−0.8989	0.0651	0.079*	
C26	−0.2454 (8)	−0.7326 (7)	0.0538 (3)	0.055 (2)	
H26A	−0.1576	−0.7432	0.0471	0.066*	
C27	−0.5351 (9)	−0.9212 (8)	0.0868 (4)	0.093 (3)	
H27A	−0.4845	−0.9904	0.0840	0.139*	
H27B	−0.6185	−0.9234	0.0554	0.139*	
H27C	−0.5551	−0.9152	0.1290	0.139*	

### Atomic displacement parameters (Å^2^)

**Table:**

	*U*^11^	*U*^22^	*U*^33^	*U*^12^	*U*^13^	*U*^23^
O	0.078 (4)	0.049 (4)	0.070 (4)	−0.004 (3)	0.032 (3)	−0.014 (3)
Br1	0.0687 (6)	0.0546 (6)	0.1100 (8)	−0.0143 (5)	0.0262 (5)	−0.0154 (6)
N1	0.048 (4)	0.029 (3)	0.062 (4)	−0.009 (3)	0.026 (3)	−0.016 (3)
C1	0.045 (4)	0.031 (4)	0.068 (5)	−0.002 (4)	0.008 (4)	0.008 (4)
Br2	0.0783 (7)	0.1131 (9)	0.0545 (5)	−0.0146 (6)	0.0232 (5)	−0.0214 (6)
N2	0.055 (4)	0.042 (4)	0.073 (4)	−0.009 (3)	0.029 (3)	−0.014 (4)
C2	0.038 (4)	0.035 (5)	0.066 (5)	0.002 (4)	0.005 (4)	0.003 (4)
C3	0.063 (6)	0.043 (5)	0.076 (6)	0.006 (5)	0.018 (5)	0.012 (5)
C4	0.043 (5)	0.073 (7)	0.069 (6)	0.004 (4)	0.011 (4)	−0.026 (5)
C5	0.054 (5)	0.036 (5)	0.083 (6)	−0.002 (4)	0.034 (5)	0.001 (5)
C6	0.049 (5)	0.046 (5)	0.069 (5)	0.002 (4)	0.018 (4)	0.002 (4)
C7	0.059 (5)	0.049 (6)	0.063 (5)	0.009 (4)	0.015 (4)	−0.006 (4)
C8	0.052 (5)	0.039 (5)	0.055 (5)	−0.001 (4)	0.007 (4)	−0.002 (4)
C9	0.064 (5)	0.055 (6)	0.041 (4)	0.001 (5)	0.005 (4)	0.000 (4)
C10	0.065 (5)	0.054 (6)	0.049 (5)	0.008 (4)	0.015 (4)	0.001 (4)
C11	0.069 (5)	0.056 (5)	0.046 (5)	−0.019 (5)	0.015 (4)	−0.006 (4)
C12	0.073 (6)	0.079 (7)	0.043 (5)	−0.010 (6)	0.009 (5)	0.003 (5)
C13	0.059 (5)	0.049 (5)	0.054 (5)	−0.003 (4)	0.002 (4)	0.004 (4)
C14	0.052 (5)	0.056 (6)	0.069 (6)	−0.007 (4)	0.023 (4)	−0.002 (5)
C15	0.065 (5)	0.039 (5)	0.087 (6)	−0.006 (4)	0.035 (5)	−0.020 (5)
C16	0.087 (6)	0.045 (5)	0.076 (6)	−0.002 (5)	0.051 (5)	−0.006 (5)
C17	0.057 (5)	0.049 (6)	0.073 (6)	0.005 (4)	0.025 (5)	−0.006 (4)
C18	0.056 (5)	0.044 (5)	0.055 (5)	0.008 (4)	0.014 (4)	−0.010 (4)
C19	0.052 (5)	0.045 (5)	0.064 (5)	−0.001 (4)	0.004 (4)	−0.009 (4)
C20	0.064 (5)	0.047 (5)	0.044 (5)	−0.003 (4)	0.004 (4)	0.003 (4)
C21	0.055 (5)	0.049 (5)	0.040 (4)	0.008 (5)	0.007 (4)	−0.009 (4)
C22	0.073 (6)	0.045 (6)	0.067 (6)	0.004 (4)	0.026 (5)	−0.003 (4)
C23	0.067 (6)	0.062 (6)	0.063 (5)	0.013 (5)	0.017 (4)	0.011 (5)
C24	0.079 (6)	0.052 (6)	0.048 (5)	−0.003 (5)	0.014 (5)	0.002 (4)
C25	0.091 (7)	0.038 (5)	0.069 (6)	0.010 (5)	0.017 (5)	0.004 (5)
C26	0.065 (5)	0.047 (6)	0.053 (5)	0.008 (5)	0.013 (4)	0.007 (4)
C27	0.102 (8)	0.078 (7)	0.094 (7)	−0.022 (6)	0.009 (6)	0.015 (6)

### Geometric parameters (Å, º)

**Table:**

O—C18	1.210 (8)	C13—H13A	0.9300
Br1—C5	1.890 (7)	C14—C15	1.544 (10)
N1—C17	1.441 (8)	C14—H14A	0.9700
N1—C1	1.470 (8)	C14—H14B	0.9700
N1—C14	1.472 (8)	C15—H15A	0.9700
C1—C2	1.491 (9)	C15—H15B	0.9700
C1—C8	1.506 (9)	C16—C17	1.496 (10)
C1—H1A	0.9800	C16—H16A	0.9700
Br2—C11	1.904 (8)	C16—H16B	0.9700
N2—C18	1.370 (9)	C17—H17A	0.9700
N2—C15	1.466 (8)	C17—H17B	0.9700
N2—C16	1.466 (9)	C18—C19	1.466 (10)
C2—C3	1.373 (9)	C19—C20	1.317 (10)
C2—C7	1.374 (10)	C19—H19A	0.9300
C3—C4	1.365 (10)	C20—C21	1.468 (10)
C3—H3A	0.9300	C20—H20A	0.9300
C4—C5	1.381 (10)	C21—C22	1.380 (9)
C4—H4A	0.9300	C21—C26	1.408 (9)
C5—C6	1.389 (10)	C22—C23	1.406 (10)
C6—C7	1.385 (9)	C22—H22A	0.9300
C6—H6A	0.9300	C23—C24	1.398 (10)
C7—H7A	0.9300	C23—H23A	0.9300
C8—C9	1.370 (9)	C24—C25	1.375 (11)
C8—C13	1.380 (9)	C24—C27	1.513 (11)
C9—C10	1.364 (10)	C25—C26	1.374 (10)
C9—H9A	0.9300	C25—H25A	0.9300
C10—C11	1.380 (10)	C26—H26A	0.9300
C10—H10A	0.9300	C27—H27A	0.9600
C11—C12	1.356 (10)	C27—H27B	0.9600
C12—C13	1.354 (10)	C27—H27C	0.9600
C12—H12A	0.9300		
			
C17—N1—C1	112.1 (6)	H14A—C14—H14B	108.0
C17—N1—C14	108.3 (6)	N2—C15—C14	109.1 (6)
C1—N1—C14	107.3 (6)	N2—C15—H15A	109.9
N1—C1—C2	114.1 (6)	C14—C15—H15A	109.9
N1—C1—C8	112.3 (6)	N2—C15—H15B	109.9
C2—C1—C8	106.7 (6)	C14—C15—H15B	109.9
N1—C1—H1A	107.9	H15A—C15—H15B	108.3
C2—C1—H1A	107.9	N2—C16—C17	111.1 (6)
C8—C1—H1A	107.9	N2—C16—H16A	109.4
C18—N2—C15	127.4 (6)	C17—C16—H16A	109.4
C18—N2—C16	119.4 (6)	N2—C16—H16B	109.4
C15—N2—C16	112.3 (6)	C17—C16—H16B	109.4
C3—C2—C7	115.4 (7)	H16A—C16—H16B	108.0
C3—C2—C1	121.9 (7)	N1—C17—C16	111.1 (6)
C7—C2—C1	122.3 (7)	N1—C17—H17A	109.4
C4—C3—C2	124.3 (8)	C16—C17—H17A	109.4
C4—C3—H3A	117.9	N1—C17—H17B	109.4
C2—C3—H3A	117.9	C16—C17—H17B	109.4
C3—C4—C5	118.9 (8)	H17A—C17—H17B	108.0
C3—C4—H4A	120.6	O—C18—N2	121.2 (8)
C5—C4—H4A	120.6	O—C18—C19	121.9 (7)
C4—C5—C6	119.3 (7)	N2—C18—C19	116.8 (7)
C4—C5—Br1	119.5 (7)	C20—C19—C18	119.8 (8)
C6—C5—Br1	121.1 (6)	C20—C19—H19A	120.1
C7—C6—C5	118.7 (8)	C18—C19—H19A	120.1
C7—C6—H6A	120.7	C19—C20—C21	126.3 (8)
C5—C6—H6A	120.7	C19—C20—H20A	116.8
C2—C7—C6	123.2 (8)	C21—C20—H20A	116.8
C2—C7—H7A	118.4	C22—C21—C26	117.0 (8)
C6—C7—H7A	118.4	C22—C21—C20	124.6 (7)
C9—C8—C13	116.0 (7)	C26—C21—C20	118.4 (7)
C9—C8—C1	122.0 (7)	C21—C22—C23	121.7 (8)
C13—C8—C1	122.1 (7)	C21—C22—H22A	119.1
C10—C9—C8	124.2 (7)	C23—C22—H22A	119.1
C10—C9—H9A	117.9	C24—C23—C22	120.8 (8)
C8—C9—H9A	117.9	C24—C23—H23A	119.6
C9—C10—C11	117.2 (8)	C22—C23—H23A	119.6
C9—C10—H10A	121.4	C25—C24—C23	116.5 (8)
C11—C10—H10A	121.4	C25—C24—C27	123.5 (9)
C12—C11—C10	120.5 (8)	C23—C24—C27	120.0 (8)
C12—C11—Br2	120.5 (6)	C26—C25—C24	123.5 (8)
C10—C11—Br2	119.0 (7)	C26—C25—H25A	118.3
C13—C12—C11	120.5 (8)	C24—C25—H25A	118.3
C13—C12—H12A	119.7	C25—C26—C21	120.5 (8)
C11—C12—H12A	119.7	C25—C26—H26A	119.8
C12—C13—C8	121.6 (8)	C21—C26—H26A	119.8
C12—C13—H13A	119.2	C24—C27—H27A	109.5
C8—C13—H13A	119.2	C24—C27—H27B	109.5
N1—C14—C15	111.0 (6)	H27A—C27—H27B	109.5
N1—C14—H14A	109.4	C24—C27—H27C	109.5
C15—C14—H14A	109.4	H27A—C27—H27C	109.5
N1—C14—H14B	109.4	H27B—C27—H27C	109.5
C15—C14—H14B	109.4		
			
C17—N1—C1—C2	56.4 (8)	C1—C8—C13—C12	177.1 (7)
C14—N1—C1—C2	175.1 (6)	C17—N1—C14—C15	−60.4 (8)
C17—N1—C1—C8	177.9 (6)	C1—N1—C14—C15	178.4 (6)
C14—N1—C1—C8	−63.3 (7)	C18—N2—C15—C14	115.6 (8)
N1—C1—C2—C3	−147.5 (7)	C16—N2—C15—C14	−53.0 (9)
C8—C1—C2—C3	87.9 (8)	N1—C14—C15—N2	56.5 (8)
N1—C1—C2—C7	40.7 (10)	C18—N2—C16—C17	−115.1 (8)
C8—C1—C2—C7	−83.8 (9)	C15—N2—C16—C17	54.5 (8)
C7—C2—C3—C4	3.4 (12)	C1—N1—C17—C16	179.2 (6)
C1—C2—C3—C4	−168.9 (7)	C14—N1—C17—C16	61.0 (8)
C2—C3—C4—C5	−1.7 (12)	N2—C16—C17—N1	−58.5 (8)
C3—C4—C5—C6	−3.1 (11)	C15—N2—C18—O	−176.7 (7)
C3—C4—C5—Br1	174.6 (6)	C16—N2—C18—O	−8.8 (11)
C4—C5—C6—C7	5.9 (11)	C15—N2—C18—C19	5.8 (11)
Br1—C5—C6—C7	−171.7 (5)	C16—N2—C18—C19	173.7 (7)
C3—C2—C7—C6	−0.3 (11)	O—C18—C19—C20	24.1 (12)
C1—C2—C7—C6	172.0 (7)	N2—C18—C19—C20	−158.4 (7)
C5—C6—C7—C2	−4.3 (12)	C18—C19—C20—C21	−179.5 (7)
N1—C1—C8—C9	−44.2 (9)	C19—C20—C21—C22	7.4 (12)
C2—C1—C8—C9	81.4 (9)	C19—C20—C21—C26	−173.0 (8)
N1—C1—C8—C13	136.3 (7)	C26—C21—C22—C23	−2.0 (11)
C2—C1—C8—C13	−98.1 (8)	C20—C21—C22—C23	177.6 (7)
C13—C8—C9—C10	1.3 (11)	C21—C22—C23—C24	0.8 (12)
C1—C8—C9—C10	−178.2 (7)	C22—C23—C24—C25	0.9 (11)
C8—C9—C10—C11	−1.0 (12)	C22—C23—C24—C27	178.7 (8)
C9—C10—C11—C12	1.7 (12)	C23—C24—C25—C26	−1.6 (12)
C9—C10—C11—Br2	−179.9 (5)	C27—C24—C25—C26	−179.2 (8)
C10—C11—C12—C13	−2.9 (12)	C24—C25—C26—C21	0.5 (12)
Br2—C11—C12—C13	178.7 (6)	C22—C21—C26—C25	1.3 (11)
C11—C12—C13—C8	3.3 (12)	C20—C21—C26—C25	−178.3 (7)
C9—C8—C13—C12	−2.4 (11)		

### Hydrogen-bond geometry (Å, º)

**Table:**

*D*—H···*A*	*D*—H	H···*A*	*D*···*A*	*D*—H···*A*
C20—H20*A*···O^i^	0.93	2.55	3.445 (10)	162

Symmetry code: (i) −*x*, −*y*−1, −*z*.
